# Comparison between the AA/EPA ratio in depressed and non depressed elderly females: omega-3 fatty acid supplementation correlates with improved symptoms but does not change immunological parameters

**DOI:** 10.1186/1475-2891-11-82

**Published:** 2012-10-10

**Authors:** Angela Maria Rizzo, Paola Antonia Corsetto, Gigliola Montorfano, Annalisa Opizzi, Milena Faliva, Attilio Giacosa, Giovanni Ricevuti, Claudio Pelucchi, Bruno Berra, Mariangela Rondanelli

**Affiliations:** 1 Dipartimento di Scienze Farmacologiche e Biomolecolari, Università degli Studi di Milano, Via D. Trentacoste 2, Milan, 20134, Italy; 2 Department of Applied Health Sciences, Azienda di Servizi alla Persona (ASP) di Pavia, Università degli Studi di Pavia, Pavia, Italy; 3 Department of Gastroenterology, Policlinico di Monza, Monza, Italy; 4 Department of Internal Medicine and Therapeutics, Geriatric Section, University of Pavia, Azienda di Servizi alla Persona (ASP) di Pavia, Pavia, Italy; 5 Mario Negri Institute for Pharmacological Research, Milan, Italy

**Keywords:** Omega-3 long chain polyunsaturated fatty acids, Depressed mood, Elderly, AA/EPA, Cell membrane, Phospholipids, EPA, DHA

## Abstract

**Background:**

Depression is one of the most frequently missed diagnoses in elderly people, with obvious negative effects on quality of life. Various studies have shown that long chain omega-3 polyunsaturated fatty acids (n-3 PUFA) may be useful in its management. Our objective was to evaluate whether a supplement containing n-3 PUFA improves depressive symptoms in depressed elderly patients, and whether the blood fatty acid pattern is correlated with these changes.

**Methods:**

The severity of depressive symptoms according to the Geriatric Depression Scale (GDS), blood fatty acid composition and erythrocyte phospholipids were analyzed in 46 depressed females aged 66-95y, diagnosed with depression according to DSMIV, within the context of a randomized, double-blind, placebo-controlled trial. 22 depressed females were included in the intervention group (2.5 g/day of n-3 PUFA for 8 weeks), and 24 in the placebo group. We also measured immunological parameters (CD2, CD3, CD4, CD8, CD16, CD19 and cytokines (IL-5, IL-15).

**Results:**

The mean GDS score and AA/EPA ratio, in whole blood and RBC membrane phospholipids, were significantly lower after 2 months supplementation with n-3 PUFA. A significant correlation between the amelioration of GDS and the AA/EPA ratio with some immunological parameters, such as CD2, CD19, CD4, CD16 and the ratio CD4/CD8, was also found. Nevertheless, omega-3 supplementation did not significantly improve the studied immunological functions.

**Conclusions:**

n-3 PUFA supplementation ameliorates symptoms in elderly depression. The n-3 PUFA status may be monitored by means of the determination of whole blood AA/EPA ratio.

## Background

An unbalance in polyunsaturated fatty acid (PUFA) status is observed in various pathological conditions, especially in chronic and/or degenerative diseases associated with antioxidant system deficiency [1]. The early diagnosis of this impairment is of crucial importance, since dietary PUFA balance may contribute towards the prevention and the control of such diseases [2]. Recent scientific evidence has disclosed a correlation between the bioavailability of n-3 PUFA and the prevention or treatment of various neurological disorders, such as adult depression, schizophrenia, dyslexia, dyspraxia, autism and attention deficit/hyperactivity disorder in children (ADHD) [3], [4], [5], [6][7]. The authors of a retrospective study including 1188 elderly American subjects suggested that low levels of circulating docosahexaenoic acid (DHA) may be a significant risk factor for the development of Alzheimer dementia. They related inability to maintain a high level of DHA with a reduced capacity to synthesize DHA late in life resulting from a reduction in Δ6 desaturase activity [8]. Moreover it was postulated that abnormalities in phospholipid fatty acid composition may play a role also in psychiatric disorders, including depression, changing membrane fluidity and, consequently, influencing various neurotransmitter systems, which are believed to be related to the pathophysiology of major depression [9]. Depletion of n-3 fatty acid levels in red blood cell membranes of depressed patients has been previously reported [10][11].

Our laboratory has developed and validated a rapid method to analyze the fatty acid composition and the arachidonic acid / eicosapentanoic acid (AA/EPA) ratio in whole blood in the Italian population; the method provides a parameter that correlates directly with erythrocyte phospholipid fatty acid composition [12].

On the basis of these observations, the aim of the present double-blind placebo-controlled study was to compare the AA/EPA ratio in depressed and non depressed elderly subjects and to evaluate the role of n-3 PUFA supplementation in the treatment of depression in the elderly.

It is well known that n-3 PUFA modify cellular immune responses both in vitro and in vivo [13][14]; therefore, another aim of this study was to evaluate CD2, CD3, CD4, CD8, CD16, CD19 lymphocyte subpopulations, as well as some cytokines. In fact, numerous studies have indicated major depression as an inflammatory state with elevated levels of proinflammatory cytokines, e.g. Interleukin IL-6, IL-12, interferon (IFN)-γ [15], IL-1 and tumor necrosis factor (TNF)-α [16]. For this reason we decided to evaluate cytokines that have not yet been sufficiently studied to date, such as IL-5 and IL-15, in this study. A possible correlation between these cytokines and depression has been postulated on the basis of the increased genetic risk for major depression associated with the colony stimulating factor 2 receptor β (CSF2RB) haplotype, which encodes a protein in high affinity receptors binding IL-5 [17]. Furthermore, a gene set analysis of post-mortem brain tissue has suggested that IL-5 levels may be up-regulated in major depression [18]. Moreover, T regulatory cells may play a role in depression through down regulation of chronic inflammatory responses [19]. Based on the notion that T cells may subserve neuroprotective and anti-inflammatory functions during stress and inflammation, impaired T cell function may directly contribute to the development of depression [20]. Literature meta-analyses in this area have also revealed that depression and stress are associated through decreased lymphocytes and T cells in plasma [21].

## Material and methods

### Subjects

Eligible participants were females with age ranging between 65 and 95 years, who were recruited in a nursing home in Pavia, where they had been institutionalized for at least 3 months prior to enrolment. The protocol was approved by the Ethics Committee of the Azienda Sanitaria Locale (ASL – Local Health Unit) of Pavia and all subjects gave their written consent to the study. Data were gathered from 31st January 2006 to 31st January 2007.

Exclusion criteria included neurologic conditions other than depression and other psychiatric disorders that could have accounted for the observed psychotic disturbances. Moreover, subjects with clinically uncontrolled organic diseases or with clinically relevant laboratory abnormalities were excluded from the study. Medications for other disorders (such as hypertension, insomnia, etc.) were kept as constant as possible during the therapeutic intervention (2 months).

### Study design

The study was a single-centre, 8-week, double-blind, placebo-controlled, randomized, parallel-group study. Included subjects were randomly assigned to the treatment or the control group. Treatment group received one tablespoon of a n-3 PUFA oil orally once a day (2.5 g/day with EPA:DHA 2:1), before lunch, while the control group received one tablespoon of placebo made of a paraffin oil, with the same lemon flavour as the intervention product.

The n-3 PUFA oil was manufactured by Also S.p.A., Div. Also-Enervit, Zelbio (CO), Italy. Bottles of identical oil for each treatment group were assigned a subject number according to a coded (AB) block randomization table prepared by an independent statistician. Investigators were blinded to the randomization table, the code assignments and the procedure. As subjects were enrolled, they were assigned a progressive subject number.

Biochemical data obtained from depressed patients enrolled in the study were compared with those of a third group of healthy subjects not directly involved in the study. To this purpose, data obtained from our laboratory data base were selected taking into account, health, sex and age. Seventy-six healthy subjects, with the same median age, were selected for the comparison. The subjects were then divided into groups, taking into account the use of omega-3 supplements: 42 healthy subjects declared that they were taking 2.5 g/day of omega-3 PUFA regularly, while 34 declared that they were not taking any kind of fatty acid supplement.

For experimental purposes, subjects were indicated as: 1) Omega-3 Before (depressed subjects assigned to the treatment group before the start of omega-3 supplementation), 2) Omega-3 After (depressed subjects after omega-3 supplementation), 3) Placebo Before (depressed subjects assigned to control group before taking placebo), 4) Placebo After (depressed subjects after the use of placebo), 5) Healthy Before (healthy subjects that were not taking supplements), 6) Healthy After (healthy subjects that were taking omega-3 supplements regularly).

### Psychological evaluation

Patients were required to have a Mini-Mental State Examination (MMSE) score higher than 24 [22]. All the patients underwent a neurologic and psychiatric evaluation for the diagnosis of depression. First of all, the guidelines for diagnosis of major depressive disorder and dysthymic disorder were used by a senior psychiatrist, as reported in the *Diagnostic and Statistical Manual of Mental Disorders*, Fourth Edition (*DSM IV*) [23]. In addition to this evaluation, the presence of depressive symptoms was assessed through the use of the Geriatric Depression Scale (GDS) [24]. The GDS Long Form is a 30-item questionnaire in which participants are asked to respond by answering yes or no in reference to how they felt over the past week. Scores of 0–9 are considered normal, depending on age, education and complaints; 10–19 indicate mild depression and 20–30 indicate severe depression. No intervention other than the administration of omega-3 fatty acids was performed for depression. Subjects were asked not to change their everyday activities.

### Blood analyses

In all the recruited subjects, fasting fresh blood samples were collected by venipuncture in EDTA separator tubes and promptly applied to peripheral blood mononuclear cells (PBMC) isolation by Ficoll Density Gradient, using LSM 1077 Lymphocyte Separation Medium (PAA, Pasching, Austria) and centrifugation at 2200 rpm for 20 minutes at 20°C. The intermediate layer consisting of PBMC was washed twice in HANKS's medium (PAA) containing 0.1% BSA and 0.5 mM EDTA and twice with DMEM Buffer (PAA) containing 2 mM EDTA and 0.5% BSA. After blocking of nonspecific antibody binding, the following monoclonal antibodies and appropriate isotype controls were used for flow cytometry: CD2, CD3, CD4, CD8, CD16, and CD19 (all BD); (MBL International, Woburn, MA). Flow cytometric analysis was performed on a FACS Canto-II (BD). The acquired data were analysed by FlowJo software (TreeStar, Ashland, OR). Numbers of circulating cells were assessed by the percentage of the respective cell subset multiplied by the respective subset of absolute cell count obtained from routine blood count. Serum levels of IL-5 and IL-15 were measured by ELISA using commercial kits (BIOSOURCE International, Camarillo, CA).

### Lipid analyses

Regarding the lipid profile, the analysis was carried out blind to the subject status. Whole blood and purified erythrocyte (RBC) cell membranes were used for lipid assessment.

RBCs were separated from plasma by centrifugation and stored at −70°C until used for analysis. Cell membranes of RBC (ghosts) were prepared by lysis with hypotonic buffer (phosphate 5 mM pH 8, EDTA 0. 5 mM) and washed several times to eliminate haemoglobin residues. Ghost lipids were extracted with chloroform/methanol according to Folch [25] and fractionated by silicic acid chromatography (200–400 mesh BIORAD) into non polar lipids, glycolipids and phospholipids Red blood cell membrane phospholipids were analyzed by quantitative thin-layer chromatography to identify each species [26].

Each phospholipid species (Phosphatidyl-ethanolamine, PE; Phosphatidyl-inositols, PI; Phosphatidyl-serine, PS; Phosphatidyl-choline, PC; Sphingomyelin, SM) was further purified by HPLC-ELSD as previously described [27].

Fatty acid composition of whole blood, total RBC membrane phospholipids and of each phospholipid species purified form RBC membrane was determined by gas-chromatographic analysis. Whole blood was directly derivatized without any further purification.

The fatty acid methylesters were obtained after derivatization with sodium methoxide in methanol 3.33% w/v and injected into gas chromatograph (Agilent Technologies 6850 Series II) equipped with a flame ionization detector (FID) under the following experimental conditions: capillary column: AT Silar length 30 m, film thickness 0.25 μm. Gas carrier: helium, temperature: injector 250°C, detector 275°C, oven 50°C for 2 min, rate of 10°C min^-1^ until 200°C for 20 min. A standard mixture containing methyl ester fatty acids was injected for calibration.

### Statistical analysis

The sample size was computed on the basis of the primary end-point of the study (i.e. Geriatric Depression Scale, GDS). To detect a 20% difference in the GDS between the n-3 PUFA and the placebo groups, with an SD of the GDS equal to 4, a minimum of 23 subjects per group were needed, with alpha=0.05 and beta=0.2. Data were analysed through the use of the statistical software package SAS version 9.1 (Cary, NC, USA). To compare depressed and non depressed subjects, as well as post-treatment to baseline values, we used Student’s paired t-test or Wilcoxon signed-rank test, according to the result of the normality test. Similarly, to compare baseline values and changes from baseline between treatment and placebo groups, we used unpaired t-tests or Wilcoxon rank sum test. Normality was assessed using the Shapiro-Wilk test. Correlations between changes from baseline in GDS and AA/EPA, with a number of anthropometric, nutritional and biological covariates were tested by calculating the Spearman rank-order correlation coefficients. P-values <0.05 were considered to be statistically significant.

## Results

A total of forty-six female subjects were enrolled. Twenty-two patients were randomly allocated to the intervention group (Omega-3 group, 2.5 grams EPA:DHA 2:1), and 24 to the placebo group. Population characteristics were similar in both groups, as shown in Table 1. The omega 3 supplement was well tolerated, and there were no serious adverse events over the 8 wk of the study.

**Table 1:** Baseline characteristics of the depressed subjects

	**Omega-3 group **	**Placebo group **
N subjects studied	22	24
Age (y)	84.9±6.9	83.0±7.3
Geriatric Depression Scale (GDS) score	17.1±3.6	16.7±4.3

**Figure 1:**
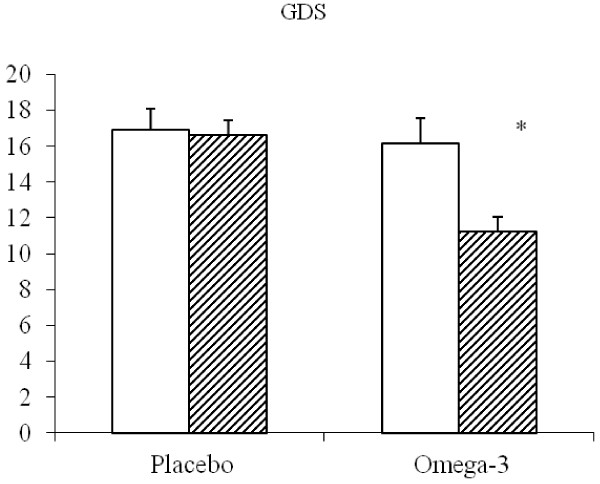
**GDS (mean±SE) in depressed elderly patients before and after supplementation with Placebo or omega-3 PUFA.** *P<0.05 After vs Before.

**Figure 2:**
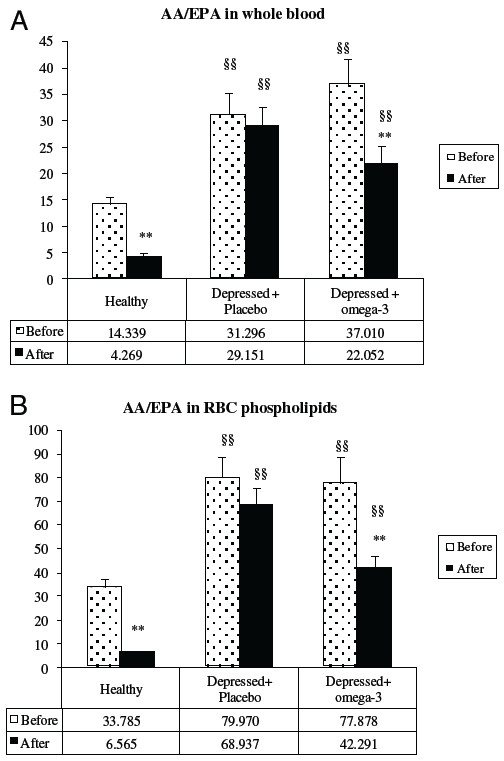
**AA/EPA ratio in whole blood (panel A) and in RBC membrane phospholipids (panel B) in healthy subjects and depressed elderly patients before and after supplementation with Placebo or omega-3 PUFA.** **P<0.01 After vs. Before; §§ P<0.01 Depressed vs. Healthy.

At baseline, the intervention group showed a mean GDS value of 17.1±3.7, while the placebo group had a mean GDS of 16.7±4.3 At the end of the study, the mean GDS score diminished significantly only in the Omega-3 group down to 11.6±4.3 (Figure 1). Figure 2 reports the AA/EPA ratio in whole blood (panel A) and in erythrocyte membrane phospholipids (panel B) of depressed subjects. For comparative purposes a group of healthy elderly subjects with the same median age who were taking (before) or not taking (after) omega-3 supplements was selected from our data-base and analyzed. At baseline, the AA/EPA ratio was significantly higher in depressed subjects than in healthy ones. At the end of the study, the AA/EPA ratio decreased significantly in the patients who had taken the omega-3 supplement (intervention group), while it did not change in the placebo group, both in whole blood or in the RBC phospholipids. Anyhow, these values appeared to be significantly higher than the AA/EPA ratio observed in both groups of healthy subjects who were or were not taking omega-3 supplements. The AA/DHA ratio in whole blood did not show significant changes after treatment (7.13±1.92 *vs* 7.01±2.36 in the omega-3 group: data not shown).

**Figure 3:**
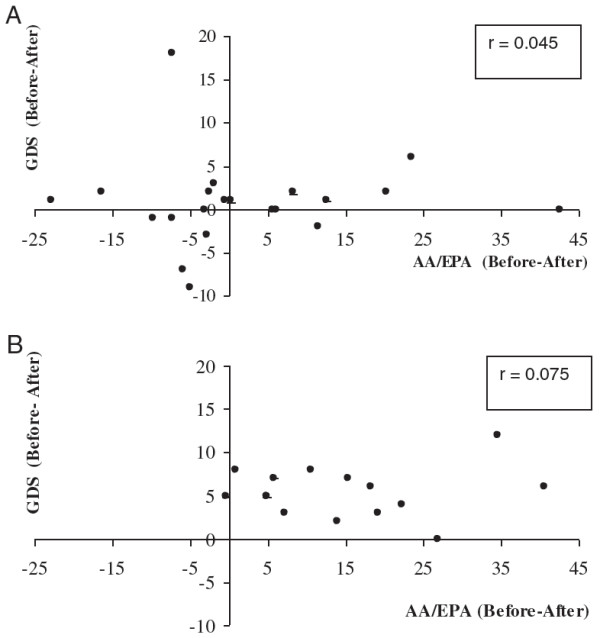
Correlations between the decrease in AA/EPA ratio (Before-After) and the change in GDS (Before-After) in depressed elderly patients treated with Placebo (panel A) and Omega-3 PUFA (panel B).

Figure 3 reports the correlation between the changes in AA/EPA ratio (Before-After) and the change in GDS (Before-After) in depressed elderly patients treated with Placebo (panel A) and n-3 PUFA (panel B). In the placebo group the data are scattered along the axes, whereas in the n-3 PUFA group all the data are positioned in the positive quadrant. This finding indicates that there is a correspondence between the amelioration of the psychological condition (as assessed by a decrease of GDS score) and the n-3 PUFA treatment (as assessed by the decrease in the whole blood AA/EPA ratio).

**Figure 4:**
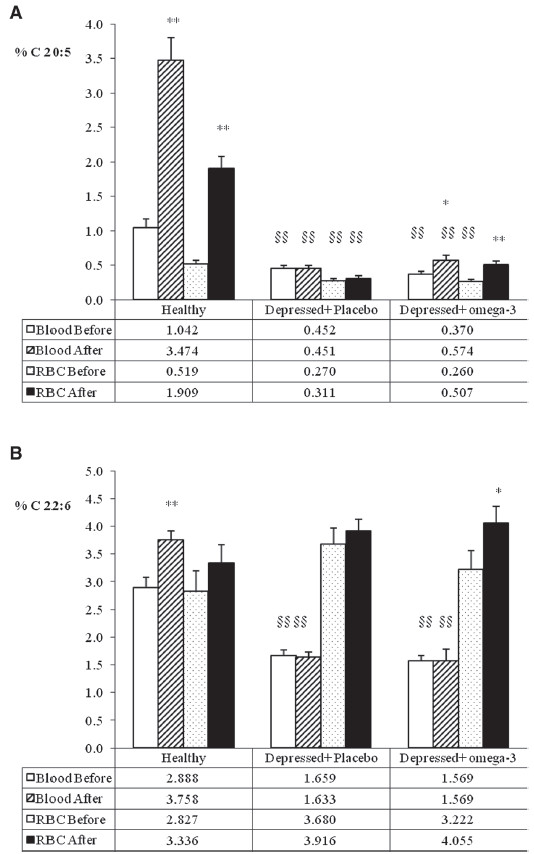
**EPA (panel A) and DHA (panel B) % concentration in whole blood and in erythrocyte membrane (RBC) in healthy subjects and depressed elderly patients before and after supplementation with placebo or omega-3 PUFA.** *P<0.05; **P<0.01 After vs. Before; §§ P<0.01 Depressed vs. Healthy before.

Figure 4 compares the EPA and DHA content (% of total fatty acids) in whole blood and RBC membranes of the healthy and depressed subjects included in the study. In the depressed elderly subjects The content of EPA (panel A) in blood and RBC membranes was significantly lower in the depressed elderly subjects than in the healthy ones. The content of EPA was increased in blood after n-3 PUFA supplementation in depressed subjects, but it did not reach the level of healthy volunteers taking omega-3. Conversely, after omega-3 supplementation the level of EPA in RBC membranes in depressed subjects reached the levels of healthy subjects that were not taking omega-3 (about 0.5%).

The level of DHA (panel B) was lower in the blood of depressed subjects than in the blood of healthy subjects; conversely, the concentration in RBC membranes did not differ when healthy and depressed subjects were compared. The DHA content of blood was not increased after supplementation, while a slightly significant increase was found in RBC membranes (from 3.22 to 4.055%).

**Figure 5:**
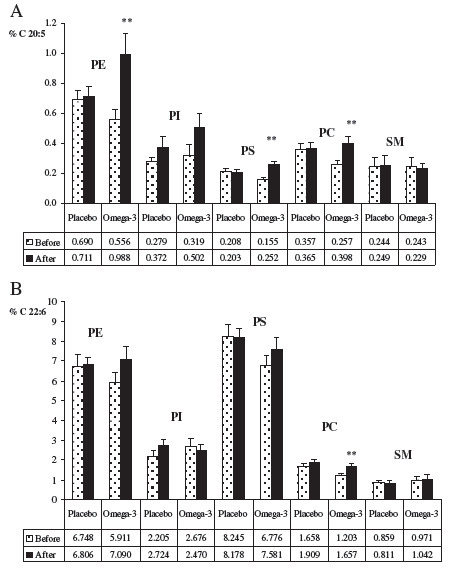
**EPA (panel A) and DHA (panel B) % concentration in specific phospholipids of erythrocyte membrane (RBC) in depressed elderly patients before and after supplementation with placebo or omega-3 PUFA.** *P<0.05; **P<0.01 After vs. Before; PE phosphatidylethanolamine, PI phosphatidylinositol; PS phosphatidylserine; PC phosphatidylcholine; SM sphingomyelin.

The phospholipid species present in erythrocyte membranes of depressed subjects were purified and analyzed in order to determine their fatty acid composition (Figure 5). EPA (panel A) was incorporated in the n-3 PUFA intervention group mainly in PE, PS and PC phospholipids. A slight increase in PI was also found, but it was not statistically significant. Conversely, DHA (panel B) was incorporated exclusively in PC, although a slight, albeit non significant increase was measured also in PE and PS.

No significant changes were found in phospholipid distribution (data not shown).

As far as immunological parameters are concerned, CD16 changed in relation to n-3 PUFA supplementation, with a significant increase in the blood of depressed subjects (Table 2).

**Table 2:** Immunological parameters: pre and post treatment immune marker comparison between the two groups of depressed subjects

	**Omega-3 group (n=22)**		**Placebo group (n=24)**		**Tests**			
	Pre-treatment	Post-treatment	Pre-treatment	Post-treatment				
	Mean (Std.dev.)	Mean (Std.dev.)	Mean (Std.dev.)	Mean (Std.dev.)	1	2	3	4
CD2	84.2 (12.2)	82.9 (12.9)	88.4 (5.5)	87.9 (8.0)				
CD19	9.0 (10.9)	10.6 (10.2)	6.2 (4.5)	10.9 (18.3)				
CD3	66.1 (12.8)	66.5 (12.9)	69.8 (8.8)	70.6 (9.6)				
CD4	41.4 (14.3)	39.1 (16.3)	44.4 (7.9)	41.4 (11.9)				
CD8	31.9 (13.4)	29.1 (13.9)	29.5 (8.1)	30.9 (9.6)				
CD16	13.0 (9.3)	16.5 (10.8)	12.4 (6.2)	15.0 (8.6)	*			
CD4/CD8	1.58 (0.96)	1.68 (1.17)	1.66 (0.85)	1.45 (0.64)				
IL-5	1.69 (0.60)	1.89 (1.28)	4.36 (8.22)	3.23 (5.90)				
IL-15	5.86 (3.88)	8.11 (6.14)	5.88 (3.32)	5.99 (5.41)				

1 Student’s paired t-test or Wilcoxon signed-rank test to compare post-treatment to baseline values within n-3 group.

2 Student’s paired t-test or Wilcoxon signed-rank test to compare post-treatment to baseline values within placebo group.

3 Unpaired t-test or Wilcoxon rank sum test to compare baseline values between n-3 and placebo group.

4 Unpaired t-test or Wilcoxon rank sum test to compare changes from baseline values between n-3 and placebo group.

* p<0.05.

Table 3 reports the correlation between changes pre and post treatment in the depressed subjects after n-3 PUFA supplementation, for all the studied parameters.

**Table 3:** Correlation (Spearman, non parametric) between changes pre/post treatment in omega-3 treated depressed subjects of the studied variables (GDS and AA/EPA) and of hematological, biochemical and immunological parameters

	**GDS**	**p**	**AA/EPA blood**	**p**	**AA/EPA membranes**	**p**
HGB	−0.16		−0.30		−0.36	
HCT	−0.08		−0.15		−0.21	
WBC	−0.13		−0.32		0.10	
Lymphocytes	−0.10		−0.15		−0.22	
RBC	0.14		−0.23		−0.02	
MCV	−0.13		−0.06		−0.03	
Plt	0.15		−0.14		−0.11	
Ferritin	−0.02		−0.17		−0.05	
Serum Iron	0.25		−0.15		−0.28	
Transferrin	−0.21		−0.03		−0.05	
Triglycerides	−0.11		−0.14		−0.16	
Total Cholesterol	−0.05		−0.39		−0.04	
HDL	−0.08		−0.15		−0.24	
Total Protein	−0.21		−0.05		−0.04	
Albumin	−0.17		−0.07		−0.31	
Creatinine	0.31		−0.02		−0.12	
Sodium	0.38		−0.12		−0.06	
Potassium	−0.11		−0.26		−0.09	
Chlorine	−0.22		−0.10		−0.12	
Calcium	−0.04		−0.04		−0.23	
Uricemia	−0.17		−0.45	*	−0.04	
Glycemia	−0.01		−0.15		−0.18	
Folic Acid	−0.32		−0.07		−0.40	
Vitamin B12	−0.15		−0.40		−0.12	
TSH	−0.03		−0.21		−0.28	
FT3	−0.56	**	−0.19		−0.36	
FT4	−0.05		−0.27		−0.14	
CD2	−0.18		−0.40		−0.45	*
CD19	−0.27		0.43	*	−0.35	
CD3	−0.48	*	−0.12		−0.26	
CD4	−0.47	*	−0.26		−0.23	
CD8	−0.21		0.18		−0.18	
CD16	−0.39		0.50	*	−0.22	
CD4/CD8	−0.48	*	−0.21		−0.12	
IL-5	−0.04		−0.01		−0.30	
IL-15	−0.20		0.45		−0.18	

* p<0.05; ** p<0.01.

A significant positive correlation was found between GDS and FT3, CD3, CD4 and CD4/CD8. The AA/EPA ratio in blood correlated positively with CD19 and CD16, while the AA/EPA ratio in membranes correlated negatively with CD2.

## Discussion

Depression is common in late life: both major and minor depression are reported in 13% of community dwelling older adults, 24% of older medical out-patients, 30% of older acute care patients and 43% of nursing home dwelling older adults [28]. Depression is often reversible with prompt and appropriate treatment. However, if left untreated, depression may result in the onset of physical, cognitive and social impairment, as well as delayed recovery from medical illness and surgery, increased health care utilization and suicide. A wealth of evidence indicates that consumption of fish or dietary fish oils containing long-chain n-3 PUFA, is associated with cardiovascular benefits, including a reduction in circulating triglycerides and reduced mortality from coronary heart disease [29]. Moreover, n-3 PUFA have been proposed as potential treatment of depressive disorders [30]. Lower concentrations of n-3 PUFA are associated not only with cognitive impairment and dementia, but also with depression, a potential risk factor for cognitive decline [31]. Our data clearly demonstrate that elderly depression is characterized by very low levels of n-3 PUFA, in particular of EPA, in RBC membranes compared to healthy subjects. In this study, the supplementation with 2.5 grams/day of n-3 PUFA for eight weeks with an EPA/DHA ratio 2:1 reduced the GDS of the depressed subjects. This positive result appears to be related to the chosen supplementation protocol used in the study. As a matter of fact, the inconsistence of the outcomes of different meta-analyses has been ascribed to differences in dose and type of the n-3 PUFA supplements used for the treatment of major depression (MDD). Martins et al. [32] reviewed recently the effects of n-3 PUFA dosages from 1 g to 6 g/day as well as of EPA and DHA alone or in different combinations of TG or ethyl esters for different treatment periods. These authors concluded that n-3 PUFA supplementation is beneficial in adult patients with MDD, but that its effect is strongly dependent upon the EPA content of the regimen. Thus, studies employing regimens containing more than 60% of EPA showed a highly significant pooled standard mean difference estimate, whereas those containing less than 60% of EPA did not .

Omega-3 supplements used at different EPA:DHA ratios, such as 1.2:1 at higher doses, were able to increase both EPA and DHA concentration in RBC membranes in adults affected by moderate hypertriglyceridemia [33].

In this study, daily supplementation with 2.5 g/day of n-3 PUFA with EPA/DHA content of 2:1 appeared able to restore only EPA concentration in RBC membrane to normal values, while the blood EPA and DHA concentrations after supplementation did not increase to the value observed in healthy subjects. Moreover, in this study, after supplementation in depressed subjects EPA appeared to be enriched in PE, PS and PC of RBC phospholipids, while DHA selectively increased in PC. This may be relevant for the production of PUFA metabolites from phospholipids, arising from second messengers and intracellular signals.

In fact n-3 PUFA, especially DHA, are increasingly being recognized as reservoirs of lipid messengers for neuronal cells, [34]; [35]. Specific precursors are cleaved from membrane phospholipids, in particular from PC, upon stimulation by neurotransmitters, neurotrophic factors, cytokines, membrane depolarization, ion channel activation. The lipid messengers, such as neuroprotectins, that originate from the cleaved DHA regulate and interact with multiple signalling cascades, contributing to the growth, differentiation, function, protection and repair of the nervous system [36]. From this point of view, our data demonstrate that in depressed subjects the level of DHA is preserved in RBC membranes while it is decreased in whole blood. After n-3 PUFA supplementation, the incorporation of DHA exclusively in PC of membrane phospholipids may favor the production of DHA metabolites.

The incorporation of PUFA in the phospholipid's sn*-*2 acyl chain differs, depending on the brain area considered. For instance, it has been demonstrated that the cortex (where DHA levels are very high) is particularly affected by n-3 PUFA deficiencies [37]. In particular, prolonged dietary deficiency leads to decrements of DHA in the order of 40% in the frontal cortex and striatum of mice. DHA brain deficiency has important neurological consequences [38]; for instance, when maintained on an n-3 deficient diet, rats or mice exhibit poorer performance in the Y-maze [39] and spatial learning [37] tests. These alterations can be corrected by feeding rodents adequate amounts of omega-3 fatty acids [40]. Moreover, the action of mood stabilizers, such as lithium, carbamazepine, sodium valproate, or lamotrigine, in bipolar disorders has been linked to arachidonic acid turnover in brain phospholipids [41]. Lithium has proved to reduce AA, but not DHA turnover selectively in phosphatidylinositol, phosphatidylcholine, and phosphatidylethanolamine [42].

Our data might reflect what is really happening in the brain of depressed subjects, indicating a n-3 PUFA deficiency, which is partially corrected after supplementation.

When n-3 PUFA concentration is low, abnormal function and metabolism of various neurotransmitters has been observed in addition to reduced activity of receptors associated with G-proteins and ionic channels. For instance, n-3 PUFA facilitate hippocampal synaptic transmission by targeting presynaptic nicotinic Ach receptors under the influence of protein kinase C (PKC) [43]. Therefore, a reduction of DHA and EPA body storage may limit the learning process and may imply derangement of cerebral activity [44].

Various experimental studies demonstrate a positive correlation between decrease in n-3 PUFA and abnormal activity of dopaminergic, noradrenergic and serotoninergic systems [45]. In vitro, serotonin reuptake, a site of action for many antidepressants, is increased by n-3 PUFA [46]. In animal models, chronic dietary n-3 fatty acid deficiency is associated with increased extracellular serotonin (5-HT) concentrations, increased levels of 5-hydroxyindoleacetic acid (5-HIAA) and increase in 5-HIAA/5-HT ratio in the brain [47]. Moreover, preclinical data provide evidence supporting a functional link between n-3 PUFA deficiency and increased central 5-HT turnover, [48]; [49]. It has also been shown that n-3 PUFA modulates the expression of dopamine and serotonin receptors [50]. These observations are relevant, because it is well known that most depression events are associated with impairment of the serotoninergic and –at least in part- of the adrenergic system [51]. Recent data on animal models demonstrate that supplementation with n-3 PUFA produces antidepressant effects mediated by an increase in serotonergic neurotransmission, particularly in the hippocampus [52].

As regards immunological parameters, to our knowledge this is the first study that examines the relationship between n-3 PUFA levels and immunological parameters i.e. CD2, CD3, CD4, CD8, CD16, CD19 lymphocyte subpopulations and IL-5 and IL-15, in depressed elderly subjects, who received either n-3 PUFA supplements or placebo.

It is known that n-3 PUFA deficiency impairs cellular aspects of the immune response [53] and a combination of clinical, animal and in vitro studies suggest that consumption of n-3 PUFA has potential therapeutic effects in the treatment of inflammatory and autoimmune diseases [54], although the results are conflicting.

In the present study, no differences were found either in the intervention or in the placebo group, when pre and post treatment immunological indices were compared, with the exception of the increase in CD16 lymphocyte subpopulation in the n-3 PUFA group. Data from animal models and humans [55] have indicated that monocyte subsets considerably change with age. Actually it appears that CD16 monocytes can be crucial players in infection and inflammation, but the formal demonstration of their role in these pathological conditions will require selective elimination of these cells in vivo [56].

A previous study indicated that the circulating monocyte pool dynamically changes during ageing in humans. The expansion of the non-classical CD14^+^CD16^+^ subtype, alterations of surface protein and chemokine receptor expression, as well as circulating monocyte-related chemokines, likely allow the preservation of monocyte pool function throughout the majority of adulthood [57].

In this study, the correlation among immunological parameters, GDS score and AA/EPA ratio in blood and membrane, was surprisingly modest. We speculate that age-related immune imbalance might be the cause of these poor correlations, as already suggested by other authors [^62^]. Therefore, further studies are needed in the field of n-3 PUFA and immunity in elderly subjects.

## Conclusion

In conclusion, this study confirms the positive effects of n-3 PUFA supplementation in the treatment of elderly depression. It also demonstrates that n-3 PUFA status may be monitored by means of the determination of whole blood AA/EPA ratio.

## Abbreviations

PUFA: Polyunsaturated fatty acids; AA 20:4 n-6: Arachidonic acid; EPA 20:5 n-3: Eicosapentaenoic acid; DHA 22:6 n-3: Docosahexaenoic acid; GDS: Geriatric depression scale; RBC: Erythrocyte; PE: Phosphatidyl-ethanolamine; PI: Phosphatidyl-inositols; PS: Phosphatidyl-serine; PC: Phosphatidyl-choline; SM: Sphingomyelin.

## Competing interests

The authors declare that the n-3 PUFA supplement was provided by Also-Enervit SPA.

## Authors' contributions

AMR and MR conceived and designed the study, performed analysis and interpretation of data and drafted the manuscript. PAC and GM performed lipid analysis; AO and MF followed the patients during the study; GR was responsible for the psychological evaluation; AG and BB performed interpretation of data and helped in drafting the manuscript, CP performed statistical analyses. All authors read and approved the final manuscript.
